# Noise Suppression of Nitrogen-Vacancy Magnetometer in Lock-In Detection Method by Using Common Mode Rejection

**DOI:** 10.3390/mi14101823

**Published:** 2023-09-24

**Authors:** Yang Li, Doudou Zheng, Zhenhua Liu, Hui Wang, Yankang Liu, Chenyu Hou, Hao Guo, Zhonghao Li, Yashuhiro Sugawara, Jun Tang, Zongmin Ma, Jun Liu

**Affiliations:** 1 State Key Laboratory of Dynamic Measurement Technology, North University of China, Taiyuan 030051, China; liyang20160506@163.com (Y.L.); zhengddtit@163.com (D.Z.); nuistlzh@163.com (Z.L.); b20220621@st.nuc.edu.cn (H.W.); hcy210014@163.com (C.H.); guohaonuc@163.com (H.G.); lizh@nuc.edu.cn (Z.L.); tangjun@nuc.edu.cn (J.T.); 2 School of Instrument and Electronics, North University of China, Taiyuan 030051, China; sugawara@nuc.edu.cn; 3 Department of Applied Physics, Graduate School of Engineering, Osaka University, 2-1 Yamada-oka, Suita 565-0871, Japan

**Keywords:** NV center, common mode rejection, bandwidth, magnetic field resolution

## Abstract

Nitrogen-vacancy (NV) centers in diamonds are promising solid-state magnetic sensors with potential applications in power systems, geomagnetic navigation, and diamond NV color center current transformers, in which both high bandwidth and high magnetic field resolution are required. The wide bandwidth requirement often necessitates high laser power, but this induces significant laser fluctuation noise that affects the detection magnetic field resolution severely. Therefore, enhancement of the magnetic field resolution of wide-bandwidth NV center magnetic sensors is highly important because of the reciprocal effects of the bandwidth and magnetic field resolution. In this article, we develop a common mode rejection (CMR) model to eliminate the laser noise effectively. The simulation results show that the noise level of the light-detected magnetic resonance signal is significantly reduced by a factor of 6.2 after applying the CMR technique. After optimization of the laser power and modulation frequency parameters, the optimal system bandwidth was found to be 75 Hz. Simultaneously, the system’s detection magnetic field resolution was enhanced significantly, increasing from 4.49 nT/Hz^1/2^ to 790.8 pT/Hz^1/2^, which represents an improvement of nearly 5.7 times. This wide-bandwidth, high-magnetic field resolution NV color center magnetic sensor will have applications including power systems, geomagnetic navigation, and diamond NV color center current transformers.

## 1. Introduction

NV centers have gathered significant research interest because of their electron spin properties, which enable polarization, manipulation, and readout capabilities. The NV centers in diamond hold considerable promise for a variety of applications, including magnetic field measurements [1], electric field measurements [2], temperature measurements [3], bioimaging [4], medical imaging [5], quantum registers [6], and quantum computing [7]. In these different application scenarios, the sensors focus on different metrics, including magnetic field resolution, the magnetic field detection range, the measurement bandwidth, the operating temperature, and power consumption. NV center magnetic sensors, with their wide bandwidths and high magnetic field resolution, are often required for use in fields such as power systems, geomagnetic navigation systems, and diamond NV color center current transformers. Researchers such as Hatano et al. developed high-magnetic field resolution diamond quantum sensors to measure the battery currents of electric vehicles. Their research indicated that the magnetic field of a 2 mm wide and 20 mm thick battery busbar is approximately 100 nT at a distance of 1 mm, and this field can be detected using an NV magnetic sensor with a bandwidth of 1 kHz and magnetic field resolution of 3.5 nT/Hz^1/2^ [8]. The United States Naval Academy used NV color center vector magnetometers to calibrate geomagnetic anomalies, with key device performance indicators that include magnetic field resolution of 200 pT/Hz^1/2^ and a dynamic range of ±120 μT [9]. Zhao Long et al. utilized the ultra-high accuracy as well as long-term stability of NV center current sensors in the field of current transformers. And the nonlinear effect of NV current sensors under high current, which linear effect limits the application of current sensors, was simulated, and improvements were proposed. [10]. Arai et al. from the Tokyo Institute of Technology used NV color center magnetometers to measure the cardiac magnetic signals in living mouse specimens for the first time. The bandwidth of the mouse’s heart signal is 200 Hz, which requires the NV magnetometer to have a detection bandwidth of more than 200 Hz and a magnetic field resolution of 140 pT/Hz^1/2^ [11].

To achieve the high measurement bandwidth required, high laser power is typically needed to excite the NV centers [12]. Because of the limitations of the laser sources themselves, the use of high laser power can result in significant laser fluctuation noise. Xie et al. used noise cancellation to mitigate the effects of the laser fluctuation noise, thereby improving the magnetic field resolution by an order of magnitude to achieve an optimal magnetic field resolution of 2.03 nT/Hz^1/2^ [13]. Wolf et al. mitigated the optical noise caused by the high laser power required in pulse spin echo measurements by increasing the correlation time to exceed the laser pulse duration, which resulted in a final magnetic field magnetic field resolution of 0.9 pT/Hz^1/2^ [14]. Zhang et al. minimized the gain of the optical noise to the signal noise at different frequencies in the existing differential method by the addition of a first-order compensation term. This improved method doubled the optical noise suppression capability within the measurement bandwidth [15]. In addition, for continuous waves, the gradient differential method can be used, but this method can only be used to measure magnetic gradients [16].

However, the magnetic measurement magnetic field resolution of most currently available NV center-based magnetic sensors is primarily focused on that of static magnetic fields. Because of the interplay between the bandwidth and magnetic field resolution metrics, when the system has high magnetic field resolution, it results in a reduced capability to respond to changing magnetic fields and thereby reduces the bandwidth metric. There has been a lack of further reports on wide-bandwidth, high-magnetic field resolution NV center magnetic sensors in the literature to date. In this article, the CMR model is established to demonstrate that subtraction of the laser and fluorescence signals eliminates the high-frequency laser noise effectively. Furthermore, through optimization of the laser power and modulation frequency parameters, the system’s −3 dB detection bandwidth has been enhanced significantly to 75 Hz. Notably, by building upon these advancements, the system’s noise level has been reduced considerably by nearly 5.5 times. Simultaneously, the system’s detection magnetic field resolution was ultimately enhanced to 790.8 pT/Hz^1/2^. This integrated NV magnetic sensor, with its wide bandwidth and high magnetic field resolution, will be suitable for a range of applications in power systems, geomagnetic navigation, diamond NV color center current transformers, and other fields.

## 2. Theory and Experimental Setup

### 2.1. Structure of the NV

**Figure 1:**
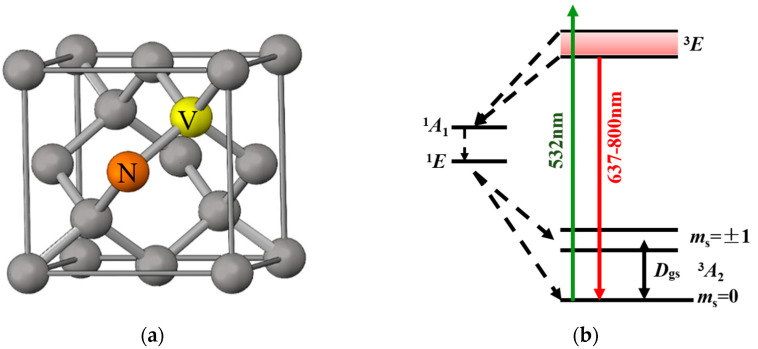
(**a**) Lattice structure of diamond NV center; (**b**) Schematic diagram of diamond NV center energy level.

The crystal structure of the NV color center is illustrated in Figure 1a [17]. NV centers are defect structures that form when two adjacent carbon atoms in diamond are substituted by a nitrogen atom and a vacancy. Depending on the number of electrons that they carry, NV centers in diamonds are typically classified into two states: NV^0^ [18] and NV^−^ [19]. While the NV^0^ center is electrically neutral, the NV^−^ center is created by a nitrogen atom and three neighboring carbon atoms that provide a total of five electrons, in addition to the capture of an extra free electron, which results in a negatively charged system. Negatively charged NV centers serve as excellent single-photon sources. The absorption (532 nm) and emission (600–800 nm) wavelengths of these centers both lie within the visible light spectrum, thus making them easily detectable during experiments. Consequently, the NV^−^ center is selected as the primary subject of investigation in this study. Unless explicitly stated to the contrary, all references to diamond NV centers in this article pertain to the negatively charged NV^−^. The energy level structure of the NV color center is illustrated in Figure 1b [20]. The ground state ^3^A_2_ of the NV color center is composed of a triplet system that consists of the sublevels of m_s_ = 0 and m_s_ = ±1. In the absence of an external magnetic field, the levels at both m_s_ = +1 and m_s_ = −1 are degenerate. At room temperature, a natural splitting frequency of *D* = 2.87 GHz exists between m_s_ = 0 and m_s_ = ±1. The corresponding excited state ^3^E also features a zero-field splitting frequency of *D* = 1.42 GHz (which is not depicted in detail in the figure for the sake of energy level structure simplification). Additionally, a metastable state designated ^1^A_1_ is present between the excited state and the ground state. The existence of this metastable state imparts distinctive effects on the photoluminescence characteristics of the NV color center.

### 2.2. Common Mode Rejection Model

The system noise mainly consists of laser fluctuation noise, photodiode noise, and circuit noise. Application of higher laser powers results in increased laser fluctuation noise, and it is essential to mitigate this noise. CMR technique can be used to address laser fluctuation noise. The fluorescence signal shows a Lorentzian shape, which can be described using the following mathematical formula:


(1) $$S(f)=S(\infty )\cdot [1-\frac{C\beta^{2}}{f^{2}+\beta^{2}}]$$

The MW signal by modulating $f_{0}$ at frequency $f_{ref}$ with depth $f_{Dev}$ produce a frequency f given by

(2) $$f=f_{0}+f_{Dev}\cdot \cos\left(2\pi \cdot f_{ref}\cdot t\right).$$

C represents the resonant signal contrast. β is given by $\beta =\frac{v}{2}$, where v corresponds to the full width at half maximum (FWHM) of the resonant signal.

If S(∞)=i+σ(t), $\sigma (t)={\sigma (t)}_{HF}+{\sigma (t)}_{LF}$, where i is the fluorescence intensity, ${\sigma (t)}_{HF}$ denotes the high-frequency laser fluctuation noise, and ${\sigma (t)}_{LF}$ stands for the low-frequency laser fluctuation noise. When f=0, S(0)=[i+σ(t)]⋅(1−C). The modulated fluorescence signal is given by

(3) $$\frac{dS(f)}{df}=\frac{d[i+\sigma (t)]}{df}-\frac{d[i+\sigma (t)]}{df}\frac{C\gamma^{2}}{f^{2}+\gamma^{2}}+[i+\sigma (t)]\frac{2C\gamma^{2}\cdot f}{{\left(f^{2}+\gamma^{2}\right)}^{2}}$$

When f≪γ and the time-varying laser power is neglected, the first two terms of the Taylor expansion of the modulated fluorescence signal are given as follows:

(4) $$S(f)=a[i+\sigma (t)]\cdot (1-C)+k\%[i+\sigma (t)]\frac{2C\cdot f_{Dev}\cdot \cos(2\pi \cdot f_{ref}t+\varphi )}{\beta^{2}}$$

where a denotes the laser fluorescence conversion efficiency. After heterodyne filtering, where the mixed signal is sin(ωt+θ), we obtain:

(5) $$S(f)=a\sigma_{HF}(1-C)\sin(\omega t+\theta )+(i+\sigma_{LF})A_{\Delta }.$$

Here, $A_{\Delta }$ is a function with Δ as its independent variable, and its curve represents the optically detected magnetic resonance (ODMR) signal. The lase signal, when shot noise and circuit noise are neglected, can be represented by the following formula:

(6) Y=i+σ(t)

where the signal entering the PD reference after passing through the attenuator is $Y^{\prime }=b[i+\sigma (t)]$. The laser signal after heterodyne filtering is given by

(7) $$Y^{\prime \prime }=b+{\sigma (t)}_{HF}\cdot \sin(\omega t+\theta ).$$

The normalized signals from the two paths are then subtracted to obtain the signal:

(8) $$Z=(i+\sigma_{LF})A_{\Delta }$$

**Figure 2:**
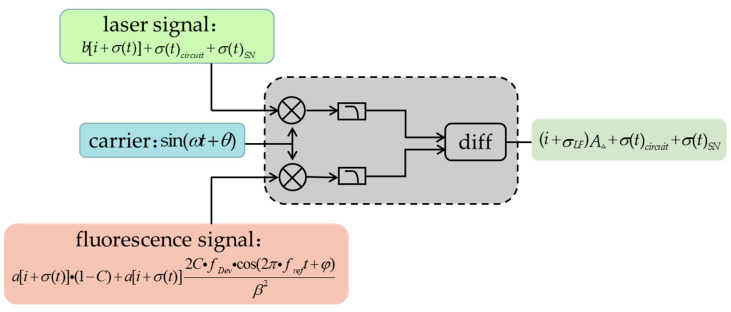
CMR technology model.

Figure 2 shows the model diagram for the CMR method.

From Equation (3), When Δ ≪ *γ*,

(9) $$\frac{dS(f)}{df}\approx \frac{d[i+\sigma (t)]/dt}{df/dt}\cdot (1-C)+[i+\sigma (t)]\frac{2C\Delta }{\gamma^{2}}$$

(10) $$\frac{dS(f)}{df}\approx [i+\sigma (t)]\frac{2Cf}{\gamma^{2}}$$

When *i* is constant, after phase-locked demodulation and low-pass filtering, the laser noise is dominated by the laser brought about by the DC component near the σ(t)·(1−C) in the $f_{ref}$ range and the RIN (laser intensity fluctuation) broadband noise that is very strongly correlated with the laser. The proportion of low-frequency noise is only a few tenths of the former at the ODMR demodulated signal pole. So, the phase cancellation noise reduction mainly targets the broadband noise with RIN around  $f_{ref}$. It is essential to note here that the fluorescence signal comprises not only the intensity fluctuation noise but also additional noise components, including the circuit noise and the shot noise. To mitigate the fluctuation noise in the fluorescence signal, it is necessary to optimize the fluorescence collection efficiency and reduce the contributions of the other noise components in the signal. Additionally, enhancement of the correlation between the reference laser signal and the fluorescence signal is essential. These actions allow effective noise cancellation to be achieved. Otherwise, direct subtraction of two signals with low correlation may lead to signal degradation.

**Figure 3:**
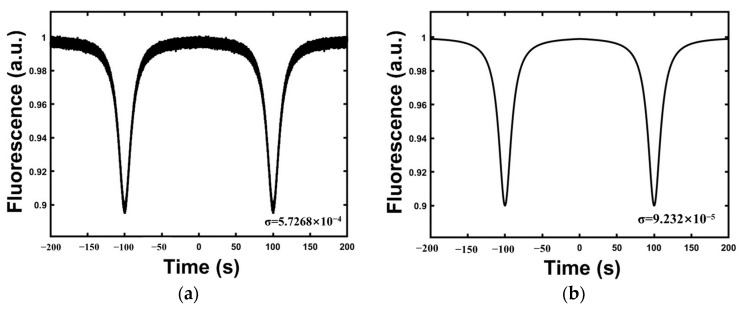
(**a**) ODMR simulation before CMR; (**b**) ODMR simulation after CMR.

By Lorentzian fitting of the fluorescence signal (see Equation (1)), we simulated the ODMR signal before and after CMR. It is assumed that the contrast C=0.1, the FWHM v=100, and the amplitude of the two signals into the PD reference and signal ends are subtracted by less than 1 mV. The simulation results are presented in Figure 3a,b. Additionally, we compared the variances before and after CMR (values indicated by σ in Figure 2) representing the signal noise in each case. Notably, after CMR, the variance was reduced by 6.2 times, thus demonstrating the substantial noise reduction effect of the process. These findings highlight the effectiveness of CMR for use in noise suppression. Consequently, when CMR is applied to laser noise suppression, precise adjustment of the attenuator used to control the laser signal amplitude is critical. This adjustment ensures that the variances of the two signals are as closely matched as possible, thereby enabling optimal noise cancellation.

### 2.3. Experimental Setup

**Figure 4:**
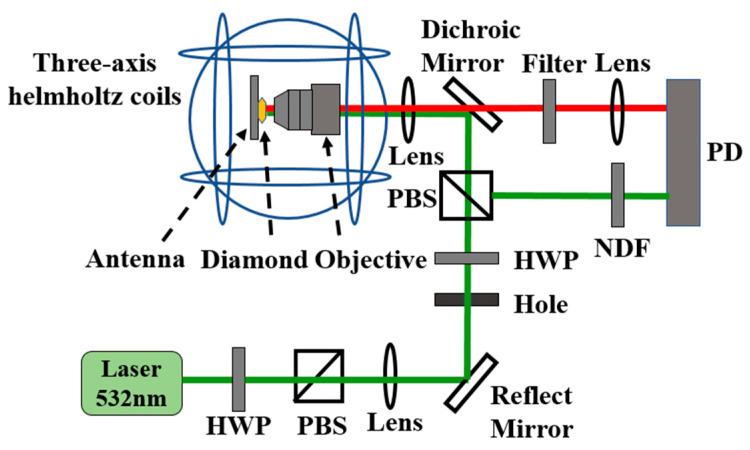
Schematic of the experimental setup.

The experimental system used in this study is depicted in Figure 4. The entire experiment was performed at room temperature. The beam output from the laser (MW-GL-532/10,000 mW 160041, Cnilaser, Changchun, China) passes through a polarizing beam splitter (PBS), with a portion of the beam being focused on the diamond surface via a 60× objective lens (OPLNFL60X, numerical aperture NA = 0.9, Thorlabs, Newton, NJ, USA). The remaining portion of the laser beam is directed to the reference end of a balanced photodetector (PDB210 A/M, Thorlabs) through the beam splitter to counteract the laser intensity noise. The NV centers are excited simultaneously by the resonant microwave field and the focused laser beam. Following this excitation, the red fluorescence emitted by the NV color centers is guided toward the signal end of the balanced photodetector through a long-pass filter. Signal subtraction is then accomplished between the reference and signal ends of the balanced photodetector, resulting in noise reduction. On acquisition of the ODMR signal of the diamond NV color centers, a low-frequency modulation signal is applied to the corresponding microwave signal at the maximum slope point in the ODMR signal, which is then demodulated using a lock-in amplifier (LIA, HF2LI 50 MHz, Zurich Instruments, Zurich, Switzerland).

The diamond sample used in our study was produced via the high-pressure high-temperature (HPHT) method by Sumitomo Electric Co., Ltd, Osaka, Japan. This sample has dimensions of 3 × 2.5 × 1 mm^3^, a nitrogen concentration of 100 ppm, and a surface polishing direction of <110>. The sample was exposed to 10 MeV electron irradiation for 4 h and was subsequently annealed at 850 °C for 2 h. The resulting NV concentration in this diamond was approximately 3.32 ppm.

## 3. Results and Discussion

### 3.1. Bandwidth

After the overall performance of the system is improved by the phase cancellation noise reduction system, we optimize the bandwidth of the system in terms of laser power and modulation frequency. The system bandwidth, which is an essential performance metric, is defined as the frequency range over which the system exhibits a response amplitude greater than −3 dB. A larger bandwidth corresponds to a higher system response speed. The frequency response is commonly expressed in logarithmic units known as decibels (dB) [11], where

(11) $$R_{dB}(f)=20\log_{10}R(f)/R_{r}$$

In this expression, $R_{dB}(f)$ represents the frequency response in dB, f denotes the corresponding frequency, and $R_{r}$ represents the reference amplitude.

**Figure 5:**
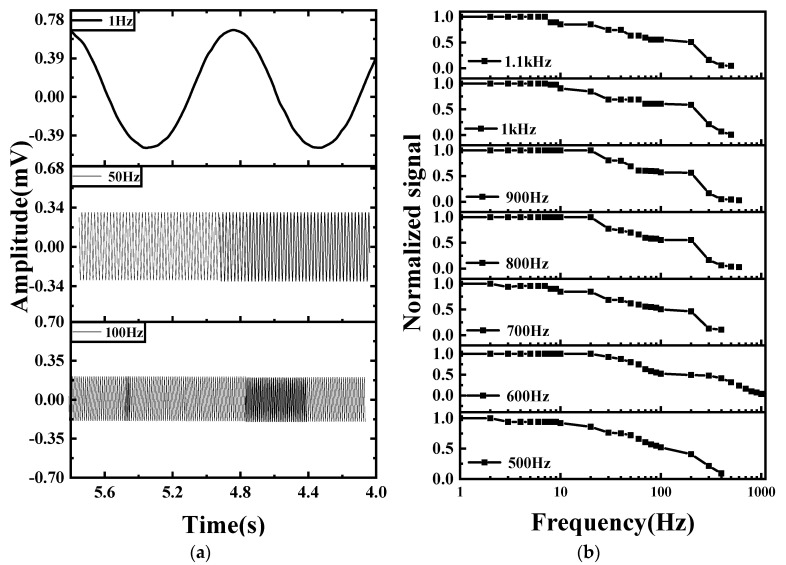

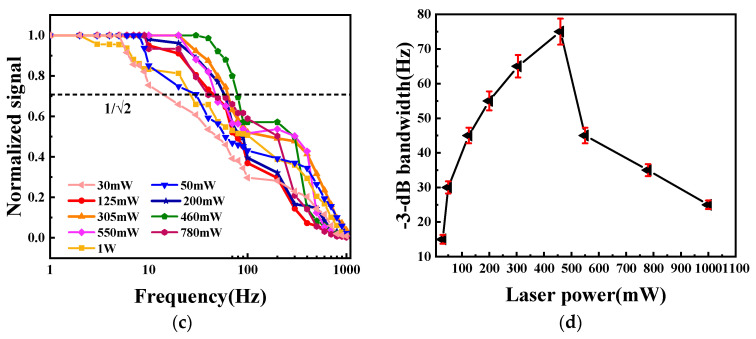
(**a**) When the laser power is constant and the frequency of b(t) is set to values of 1 Hz, 50 Hz, and 100 Hz, the amplitude of v(t) is shown as a function of time at each frequency; (**b**) The detection bandwidth at different modulation frequencies for a given amount of laser power.; (**c**) experimental measurements of the detection bandwidth under various laser powers; (**d**) −3 dB detection bandwidth as a function of the laser power.

We applied an alternating magnetic field *b*(*t*) to the diamond by generating a sinusoidal signal with a frequency range of 1–1000 Hz using a signal generator (Keithley 6221) through a three-axis Helmholtz coil. As shown in Figure 5a, under constant laser power conditions, the locked signal amplitude decreases to −3 dB when the frequencies of B(t) are set at 1 Hz, 50 Hz, and 100 Hz, with the amplitude of v(t) being presented as a function of time. The function B(t) is defined as Bcos(βt+φ), where B, β, and φ represent the signal amplitude, angular frequency, and phase, respectively. The sine wave v(t) is obtained by fixing the microwave source frequency at the zero-crossing point of the demodulation signal and then applying the alternating magnetic field b(t). This signal is recorded using the LIA, which acts as an oscilloscope in this context. The frequencies of b(t) and v(t) are identical, and their amplitudes have a proportional relationship. Figure 5b illustrates the optimal −3 dB bandwidth that can be achieved when the laser power is set to 305 mW and the optimal modulation frequency is 600 Hz. Figure 5c shows the measured amplitude as a function of the frequency of b(t) when the laser power is set to a range of different values. The detection bandwidth is ascertained through multiple experimental tests that enable the identification of the best detection bandwidth at each laser power level. The amplitude is normalized such that its value at the starting point is equal to 1, which serves as the reference amplitude. The frequency at which the normalized amplitude drops to 1/2^1/2^ is defined as the detection bandwidth. At any specific laser power, the normalized amplitude decreases as the frequency increases. As depicted in Figure 5d, the −3 dB bandwidth reaches a maximum when the laser power is set at 460 mW. The detection bandwidth of the experimental system at the optimal modulation frequency and the optimal laser power is 75 Hz, which signifies a five-fold enhancement when compared with previous work conducted by this research group [21].

### 3.2. Magnetic Field Resolution

**Figure 6:**
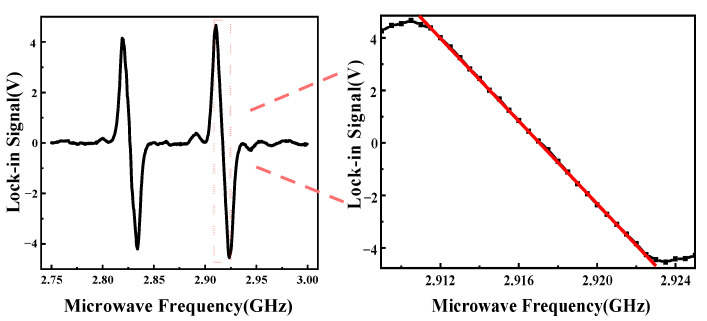
Locked signal measured via LIA, where the black line in the right-hand panel is a partial enlargement of the left-hand panel and the red line is the fitted linear interval.

We apply a uniform magnetic field of 1.5 mT near the NV center using a Helmholtz coil. A modulation frequency of $f_{ref}=500\mathrm{Hz}$ and a frequency deviation of $f_{Dev}=1\mathrm{MHz}$ were applied around the center frequency $f_{0}$ at a frequency f, where see Equation (2). Subsequently, the signal was detected using the LIA. These parameter settings were selected strategically to realize the maximum linear range and the optimal slope for the system. Specifically, the MW laser frequency was carefully selected to be at the point where the demodulated signal exhibits its highest slope. This meticulous frequency selection ensures an ideal trade-off between linearity and magnetic field resolution, thus allowing for enhanced system performance and accurate measurements to be performed using the experimental setup. We obtained the lock-in signal measured by the LIA as shown in Figure 6, where within the linear fitting interval of 2.9115–2.9225 GHz $S_{L}\sim k\left(f_{0}-f_{ref}\right)$ serves as a function of $\left(f_{0}-f_{ref}\right)$. This linear interval is regarded as the measurement range for the magnetic field’s dynamic range. The magnetic field dynamic range (ΔB) can be calculated using the formula Δf=γ·ΔB, where the frequency shift △f=11 MHz and the constant electron spin gyromagnetic ratio $\gamma =2.8\times 10^{4}\;\mathrm{MHz}/T$. The inherent dynamic range of this system is approximately 392.857 μT (±196.429 μT), which represents an improvement of 1.8 times over the range in our previous work [21].

**Figure 7:**
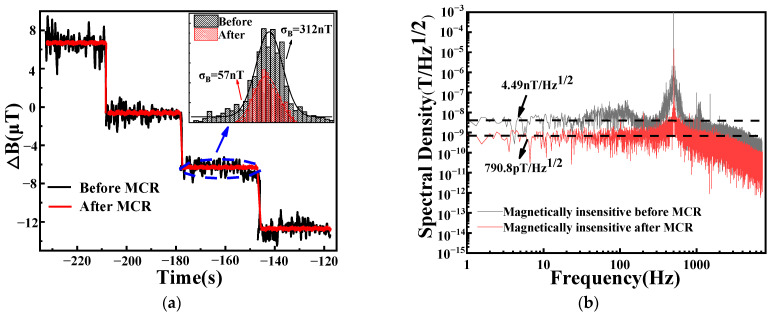
(**a**) Step responses of the system before and after use of CMR technology. The illustration shows the histogram of the magnetic field noise signals before and after application of CMR technology; (**b**) noise amplitude spectrum before and after CMR.

Figure 7a shows the step response of the system before and after use of the CMR technology. Because of the relationship between the current source and the magnetic field generated using the three-axis Helmholtz coil, the detected voltage amplitude changes with variations in the external magnetic field. The histogram depicts the noise levels before and after CMR, represented by $\sigma_{B}$, which is the standard deviation. The standard deviation $\sigma_{B}$ reflects the magnitude of the magnetic field’s magnetic field resolution directly. The magnetic field magnetic field resolution is given by $\eta =\sigma_{B}\sqrt{T}=\frac{\sigma_{B}}{\sqrt{2f_{1}}}$ [22]. A magnetic-field measurement of duration *T* (with sampling rate $F_{s}=1/T$) has a Nyquist-limited single-sided bandwidth of $f_{1}=F_{s}/2=1/(2T)$. The standard deviation of the magnetic field signal decreased from 312 nT to 57 nT after CMR, representing a reduction of nearly 5.5 times. This result indicates that the CMR technology reduced the noise level effectively and improved the magnetic field resolution.

The magnetic field resolution constraint imposed by the photon shot noise can be described using the following equation:

(12) $$\eta_{SN}\approx \frac{\Delta v}{\gamma C\sqrt{R}}$$

R can be calculated using Equation (10):

(13) $$R=\frac{P\lambda }{hc}$$

The detected fluorescence power *P* can be determined using Equation (11):

(14) $$P=\frac{U}{K\sigma }$$

In this case, Δv represents the FWHM of the ODMR signal, which is 10.5 MHz; the gyromagnetic ratio of the NV center γ is 2.8 MHz/Gs; the contrast C is 0.08; the detected fluorescence wavelength λ is 700 nm; the Planck constant h is 6.63 × 10^−34^ J·s; the speed of light c is 3 × 10^8^ m/s; the detected fluorescence voltage amplitude U is 1.8 V; the gain factor of the amplification circuit K is 500 kV/A; and the responsivity σ of the photodetector (PD) at 920 nm is 0.6 A/W. Ultimately, the magnetic field resolution limitation due to the photon shot noise is calculated to be 3.34 nT/Hz^1/2^.

In accordance with Equation (12), the measurement magnetic field resolution of our system can be computed using the noise amplitude spectral density (ASD), which is derived from the Fourier transform of the output from the LIA [23].

(15) $$\eta =\frac{ASD}{\max\left(\left|\frac{dV}{df}\right|\right)\gamma_{e}}$$

The lock-in signal for the system is displayed in Figure 6a, with the maximum slope of the demodulation signal being determined as

(16) $$\max\left|\frac{dV}{df}\right|=\left|\frac{4.66016-(-4.54492)}{2923.5-2910.5}\right|=0.708\mathrm{mV}/\mathrm{MHz}$$

As depicted in Figure 7b, according to Equation (12), the system magnetic field resolution before CMR was calculated to be 4.49 nT/Hz^1/2^. After the application of CMR technology with the optimal laser power and modulation frequency for the desired bandwidth, the magnetic field resolution improved to 790.8 pT/Hz^1/2^. The magnetic field resolution was increased by nearly 5.7 times when compared with that before CMR. Therefore, CMR technology can reduce laser intensity noise in the system effectively, especially at high laser powers, and results in a significant magnetic field resolution improvement.

The results show that the noise suppression of the NV magnetometer in the lock-in detection method by using the CMR technique is useful. The suppression of noise leads to an increase in the overall performance of the system. Although bandwidth and magnetic field resolution are in a mutually constraining relationship, we further increase the magnetic field resolution by a factor of 5.7 while maximizing the bandwidth. This method can provide a reference for other types of sensors, such as electric field sensors and acceleration sensors. In addition, the magnetic field resolution can be further improved by adding structures such as magnetic aggregation structure [24] to amplify the magnetic field and edge collection structure [25] to increase the efficiency of the fluorescent handpiece.

## 4. Conclusions

In this paper, a CMR model is established by calculating the output signals obtained by phase subtracting the signals from the signal port of the PD and the reference port. It is concluded that the phase subtraction of laser and fluorescence eliminates the laser fluctuation noise when the variance of the laser and fluorescence signals entering the PD port is kept as equal as possible. It should be noted that direct phase subtraction of two low-correlation signals degrades the signals. Simulation results on the ODMR signal show that the noise level of the ODMR signal is reduced by a factor of 6.2 after CMR. After improving the overall performance of the system, the optimum bandwidth of 75 Hz is obtained by optimizing the laser power and modulation frequency parameters, and the variance of the step signal is reduced by a factor of 5.5 after CMR by testing the step signal and obtaining the histogram of a certain section of the signal. Meanwhile, on this basis, the detection magnetic field resolution of this system is improved from 4.49 nT/Hz^1/2^ to 790.8 pT/Hz^1/2^, which is nearly 5.7 times higher than that before noise reduction. Currently, more and more experts are concerned about the effect of noise on the overall performance of the system and are doing their best to reduce the noise as close as possible to the photon scattering noise. This significant improvement paves the way toward more sensitive and precise magnetic sensing in a variety of applications. This wide-bandwidth, high-magnetic field resolution NV color center magnetic sensor will be suitable for applications in power systems, geomagnetic navigation, diamond NV color center current transformers, and many other fields.

## Data Availability

Not applicable.
